# A Systematic Review on Predisposition to Lymphoid (B and T cell) Neoplasias in Patients With Primary Immunodeficiencies and Immune Dysregulatory Disorders (Inborn Errors of Immunity)

**DOI:** 10.3389/fimmu.2019.00777

**Published:** 2019-04-16

**Authors:** Irbaz Bin Riaz, Warda Faridi, Mrinal M. Patnaik, Roshini S. Abraham

**Affiliations:** ^1^Division of Hematology, Department of Medicine, Mayo Clinic, Rochester, MN, United States; ^2^Department of Hematology, University of Arizona, Tucson, AZ, United States; ^3^Department of Pathology and Laboratory Medicine, Nationwide Children's Hospital, Columbus, OH, United States

**Keywords:** primary immunodeficiencies, B cell lymphoma, T cell lymphoma, systematic (literature) reviews, immunodeficiency

## Abstract

Primary immunodeficiencies and immune dysregulatory disorders (PIDDs; now referred to as inborn errors in immunity) are rare disorders with a prevalence of 41. 4 or 50.5 per 100,000 persons (1). The incidence of malignancy in PIDD patents is the second-highest cause of death in children as well as adults, after infection, and is higher in certain PIDDs compared to others. We performed a systematic review of the literature to identify reports of B cell and T cell neoplasias in PIDDs and clustered them based on their classification in the IUIS schema. As would be expected, higher susceptibility to malignancies are typically reported in patients with Common Variable Immunodeficiency (CVID), combined immunodeficiencies affecting cellular immunity, in particular, DNA repair defects, or in the context of impaired immune regulatory control. There is not much evidence of increased risk for cancer in patients with innate immune defects, indicating that not all types of infection or genetic susceptibility predispose equally to cancer risk. Viral infections, in particular EBV, HHV and HPV, have been shown to increase susceptibility to developing cancer, but also patients with defects in immune regulation, such as Autoimmune Lymphoproliferative Syndrome (ALPS), activated p110delta syndrome (APDS type 1) and IL-10 receptor deficiency among others have a higher incidence of neoplastic disease, particularly lymphomas. In fact, lymphomas account for two-thirds of all malignancies reported in PIDD patients (2), with either a combined immunodeficiency or DNA repair defect predominating as the underlying immune defect in one registry, or antibody deficiencies in another (3). The vast majority of lymphomas reported in the context of PIDDs are B cell lymphomas, though T cell lymphomas have been reported in a few studies, and tend to largely be associated with chromosomal breakage disorders (4) or Cartilage Hair Hypoplasia (5). There appears to be a much higher prevalence of T cell lymphomas in patients with secondary immunodeficiencies (6), though this could reflect treatment bias. We reviewed the literature and summarized the reports of B and T cell lymphoma in PIDD patients to survey the current state of knowledge in this area.

## Introduction

Monogenic and other genetic defects of the immune system, now collectively grouped as primary immunodeficiencies and immune dysregulatory disorders (PIDDs) affect various components of the immune system with susceptibility to infections, but also to autoimmunity, malignancies, and other manifestations of immune dysregulation (7–9). The number of genetically defined PIDDs is increasing with the current tally at well over 300 genes (10), and several of these are associated with an increased predisposition to developing neoplastic disease (11). Early studies have suggested a variable prevalence of malignancies in PIDDs with approximately 25% affected with cancer at the higher end of the spectrum (2). A recent large study spanning 12 years for patients enrolled in a national registry (USIDNET) revealed an age-adjusted cancer risk, as well as a gender-associated cancer risk with male patients predominating in this cohort (12). The largest proportion of risk was conferred by susceptibility to hematopoietic malignancies rather than solid tumors, in particular lymphoma. Herein, we describe a targeted literature review on the associations of B and T cell lymphomas with PIDDs, particularly with regard to specific immune defects. We will also reflect on current state of knowledge as regards to pathogenesis and management of lymphoid neoplasias in PIDDs.

## Methods and Results

**Figure 1:**
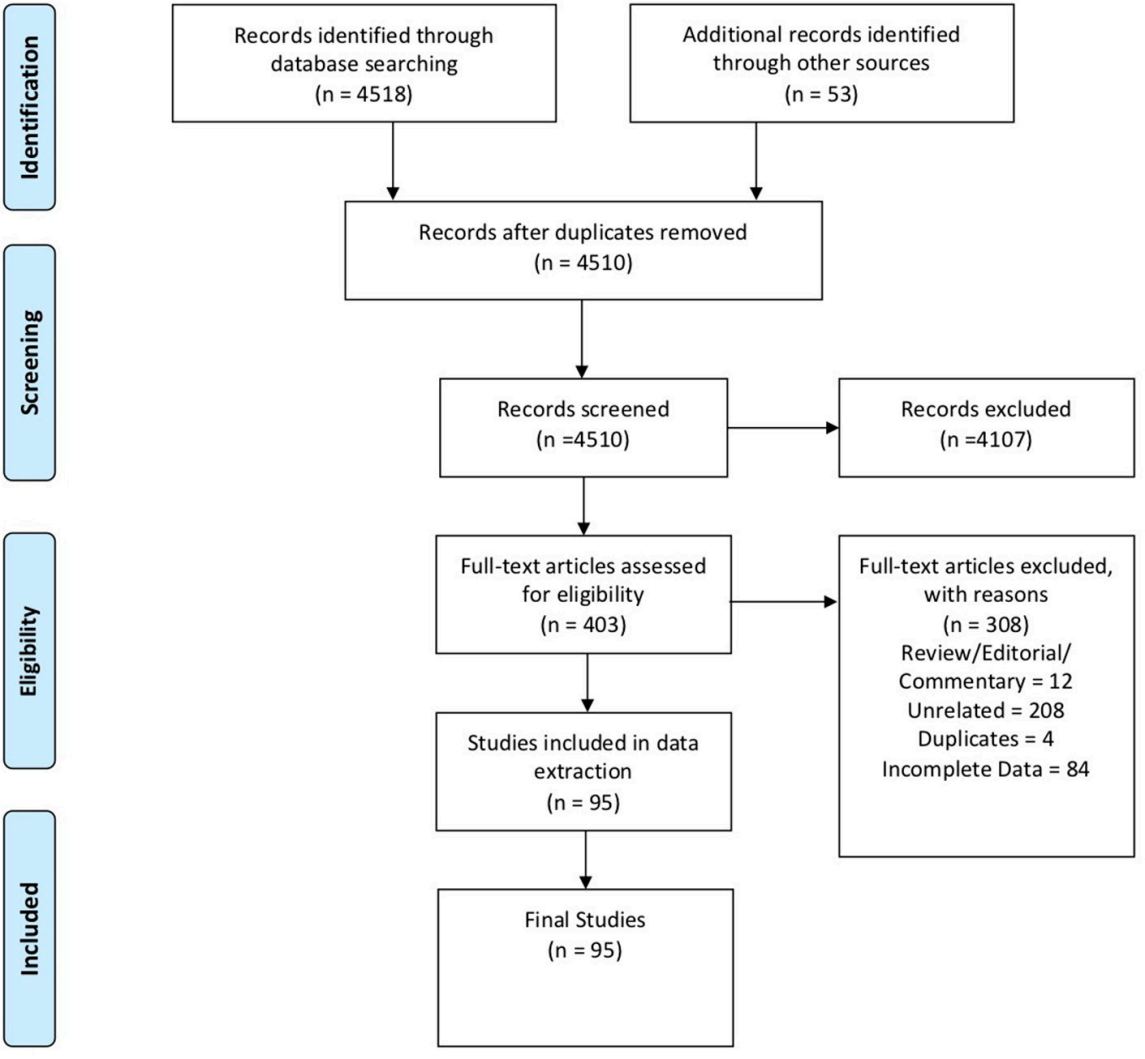
PRISMA flow diagram: The PRIMSA (Preferred Reporting Items for Systematic Reviews and Meta-Analyses) diagram details our search and selection process applied during the overview.

**Figure 2:**
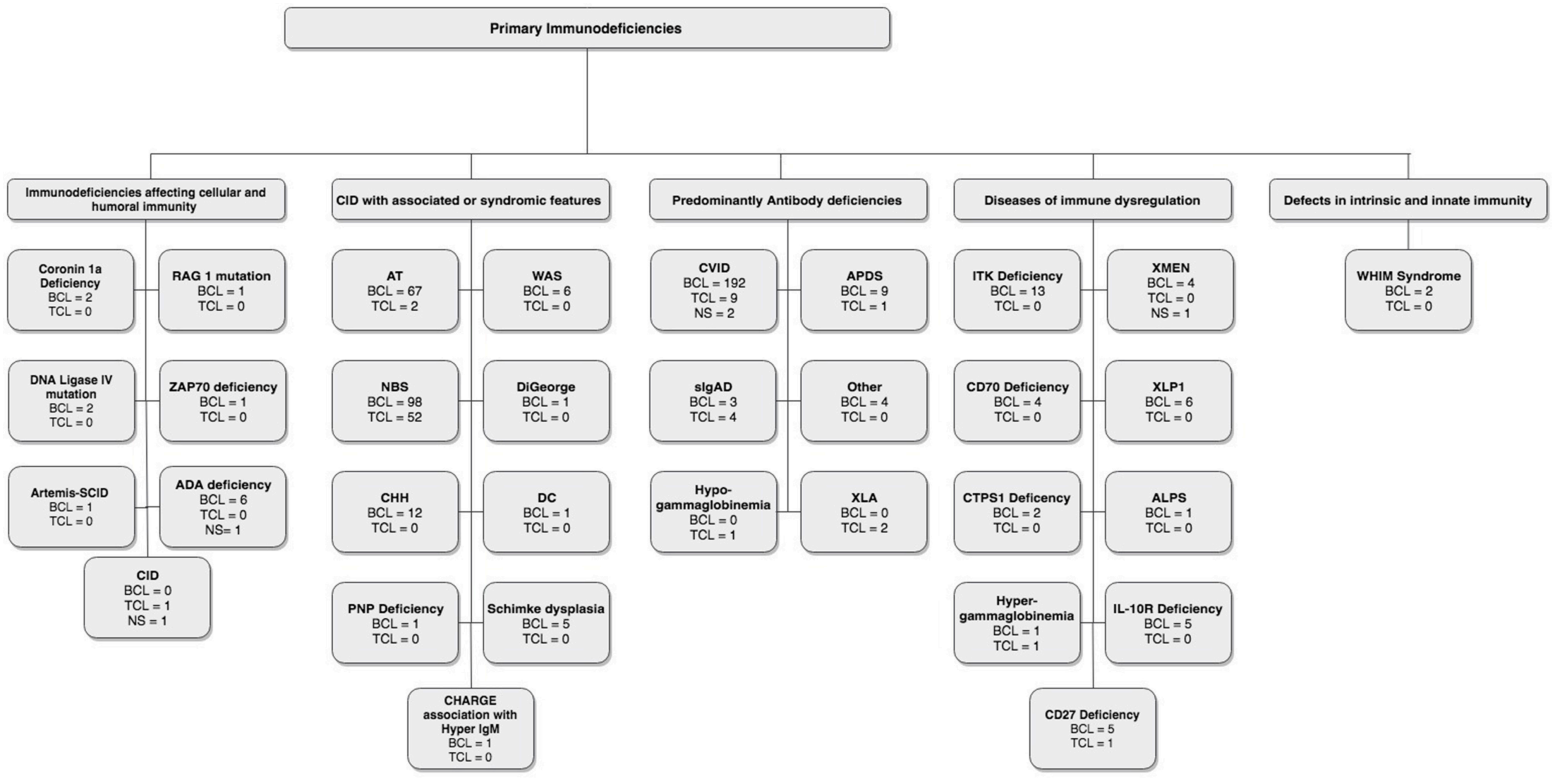
Lymphoma distribution according to IUIS classification.

In these sections, we describe the strategy to identify relevant citations gathered for this review and a summary of the results. A comprehensive search of the MEDLINE database was initially conducted from its inception to October 17th, 2018. The search was updated on February 1st, 2019. Controlled vocabulary supplemented with keywords (which included lymphoma) was used to search for individual primary immunodeficiencies as per the IUIS 2017 classification. Search results and study selection are outlined in Figure 1. Number of cases and type (B, T or unspecified) are shown in Figure 2. The cases of B cell lymphomas, T cell lymphomas and unspecified lymphomas in PIDDs are summarized in Tables 1–3 respectively.

**Table 1:** Summary of B cell lymphomas in PIDDs.

**PID**	**References**	***N***	**Proposed mechanism**	**Cancer**	**Specific mutations**	**Age/sex**	**Manifestation/course**	**Treatment**	**Outcome**
**IMMUNODEFICIENCIES AFFECTING CELLULAR AND HUMORAL IMMUNITY**									
Coronin 1a deficiency^*^	(13)	2	EBV associated	Pt1: DLBCL	Coronin-1a mutation: V134M/V134M	1/M	Left orbit mass	Chemotherapy	F/U at 11.5 y = CR
				Pt2: DLBCL	Coronin-1a mutation: V134M/V134M	0.6/F	Cervicothoracoabdominal LN and cerebral lesion	Chemotherapy	Died during induction therapy at 8.5 m of age
DNA Ligase IV mutation^*^	(14)	1	EBV associated	DLBCL	LigIV gene: M249V substitution and a 5 nucleotide del from 1,270–1,274	14/F	Progressive gingival swelling and high fever	Vincristine, cyclophosphamide, and prednisolone	Died of respiratory aspergillosis
Artemis-SCID^*^	(15)	1	DNA repair defect	BCL	Hypomorphic mutations of the Artemis gene	5/F	Liver, lung, LN, skeletal muscle involvement	Rituximab	Died of lymphoma
RAG 1 mutation^*^	(16)	1	NR	DLBCL	RAG1 gene: Allele 1 R314W^*^ Allele 2 R507W/R737H	2/F	Tumor of right tonsil	Rituximab, HSCT	CR
DNA Ligase IV mutation^*^	(17)	1	DNA repair defect EBV associated	DLBCL	Compound heterozygous for a null allele and a hypomorphic mutation in DNA LigIV	2/F	High fever, cervical LN, hepatomegaly, necrotizing mucositis.	Chemotherapy	Pt. died of aspergillosis
ZAP70 deficiency^*^	(18)	1	EBV associated	DLBCL	Zap70 Gene: c.836_837 delAT	1/F	Generalized lymphadenopathy	Vincristine, cyclophosphamide methylprednisolone	Died of DIC, multiorgan failure
ADA deficiency^*^	(19)	1	EBV associated	BCL	NR	NR	NR	NR	Dead 1 week later
ADA deficiency	(20)	1	EBV associated (suspected)	Immunoblastic plasmacytoid	NR	NR	NR	NR	Died at 4 y
ADA deficiency	(21)	1	NS	DLBCL	A83D; Exon5 splice donor site c.573 + 1G>A	10/M	6-week history of head- ache, eye deviation, and weakness.	COP + APO + 6-MP; MTX	Died after 5 m
ADA deficiency	(22)	1	Immune dysregulation	BL	Homozygous Q3X	14/F	Right hip pain and limping	Chemotherapy	CR 20 m later
ADA deficiency	(23)	1	Immune dysregulation	DLBCL	Homozygous W272X (c.815G > A)	3/F	Respiratory complaints and symptoms	BFM 2004 protocol	Died of septic shock and intracranial hemorrhage after starting treatment
ADA deficiency^*^	(24)	1	EBV associated	Plasmablastic	Homozygous 462delG	18/F	Persistent fever, multiple lymphadenopathies and bilateral periorbital edema	Rituximab; APO; and cyclophosphamide + dexamethasone	Patient died of a hemorrhagic alveolitis, 12 days after starting chemotherapy
**COMBINED IMMUNODEFICIENCIES WITH ASSOCIATED OR SYNDROMIC FEATURES**									
Ataxia Telangiectasia^*^	(25)	12	NS	HL	NS	10/8M, 4F	NR	NS	10 pts died
		38	NS	NHL	NS	9/22M, 16F	NR	NS	28 pts died
Ataxia Telangiectasia^*^	(26)	1	DNA repair defect	MZL	NS	16/M	Chronic lymphadenitis, LN at left jaw, Cervical, celiac, para-aortic	Rituximab	CR
Ataxia Telangiectasia^*^	(27)	1	DNA repair defect	HL	NR	14/M	Cervical, axillary and mediastinal LN	COPP	CR
Ataxia Telangiectasia^*^	(28)	1	EBV associated	NHL	ATM gene (exon 39 and c.6095G4A)	13/M	Waldeyer's ring, intrapulmonary, abd., tonsillar, cervical LN	Rituximab, doxorubicin, dexamethasone, cytarabine, cyclophosphamide, etoposide, vincristine, HSCT	No recurrence on 3 y FU
Wiskott Aldrich^*^	(29)	1	EBV associated DNA repair defect	DLBCL	NR	14/M	Cervical adenitis, neurological symptoms	Rituximab	Died
Wiskott Aldrich^*^	(30)	1	NS	BL	WASP gene missense mutation (105 C > T) in exon 1	12/M	Recurrent colicky abd. pain and bloody stools	Rituximab, CHOP	CR
Wiskott Aldrich^*^	(31)	1	EBV associated	DLBCL	NS	15/M	Progressive respiratory distress, night sweats	Cyclophosphamide, prednisone, radiotherapy, laser surgery	Died of lung infection
Wiskott Aldrich^*^	(32)	1	EBV associated	HL	NS	1	Pulmonary hilar LN	ABVD	CR for more than 4 y
Wiskott Aldrich^*^	(33)	1	EBV associated	NHL	NR	20/M	Lymphadenopathy, CNS, gastric wall, pulmonary lesions	Acyclovir, adenosine arabinoside	Died of sepsis
Wiskott Aldrich^*^	(34)	1	EBV associated	IL	NS	13/M	Rectosigmoid tumor	Chemotherapy, excision, L-asparaginase therapy	Died of colon perforation
DiGeorge Syndrome^*^	(35)	1	EBV associated	INHL	NS	10m/F	Hemiparesis due to cerebral mass, mediastinal LN	Untreated	Died
Cartilage hair dysplasia^*^	Unpublished	1	EBV associated	DLBCL	RMRP gene	51/M	Incidental lung nodule	R-CHOP	CR
Cartilage hair dysplasia^*^	Unpublished	1	EBV associated	MZL	RMRP gene	47/M	Fever, night sweats, lymphadenopathy	Rituximab	Under treatment with Rituximab for recurrence
Cartilage hair dysplasia	(36)	10	NS	Pt1: HL	70A > G/70A > G	20/M	NR	NR	Died in 1 m
				Pt2: NHL	70A > G/262G > T	40/M	NR	NR	Died same m.
				Pt3: NHL	dupTACTCTGTGA at 13/70A > G	45/F	NR	NR	Died in 3 m
				Pt4: NHL	70A > G/70A > G	46/F	NR	NR	Died in 6 m
				Pt5: NHL	Not tested	22/F	NR	NR	Died in 2 m
				Pt6: NHL	70A > G/70A > G	6/F	NR	NR	Died in 9 m
				Pt7: NHL	Not tested	21/M	NR	NR	Died in 1 m
				Pt8: NHL	Not tested	26/M	NR	NR	Alive 4.5 y
				Pt9: NHL	70A > G/70A > G	32/M	NR	NR	Alive 11 y
				Pt10: NHL	70A > G/70A > G	33/F	NR	NR	Alive 4.5 y
NBS	(37)	8	NS	Pt1: DLBCL	Homozygous 657del5	10/M	DLBCL at 10, 18, 23 and 26y	1st event unknown, 2nd LMB-89, 3rd R-CHOP, 4th DHAP	Alive, CR at 27 y
				Pt2: DLBCL	Homozygous 657del5	14/F	DLBCL at 14, 20y	1st event unknown, 2nd LMB-89 without MTX	Died of liver failure after CYM protocol at 20 y
				Pt3: DLBCL	Homozygous 657del5	4/F	NR	LMB-89, without CTX, followed by individualized chemotherapy	Died of disease after second individual protocol at 5 y
				Pt4: DLBCL	Homozygous 657del5	6/F	NR	LMB-89, without CTX	Died of disease at 7 y
				Pt5: DLBCL	Homozygous 657del5	11/F	NR	LMB-89 without CTX, followed by individualized chemotherapy	Died of disease at 12 y
				Pt6: DLBCL	Homozygous 657del5	23/M	Switch from DLBCL to AILT at 26y	LMB-89; for AILT: BFM-90 followed by individualized chemotherapy	Died of disease after individual protocol at 27 y
				Pt7: BL	Homozygous 657del5	9/M	Burkit like lymphoma at 9, followed by DLBCL at 10	1st LMB-89, 2nd LMB-89 followed by individualized chemotherapy and splenectomy	Alive at 16 y
				Pt8: BL	Homozygous 657del5	5/F	NR	LMB-89	PR, alive at 12
NBS	(38)	11	NS	Pt1: DLBCL	NR	9/M	NR	NHL-BFM86	CR 11 y after diagnosis
				Pt2: DLBCL	NR	15/F	B-NHL at 31 y, third malignancy Bi-linage leukemia at 34 y	NHL-BFM86 modified	Died at 34y from 3rd malignancy
				Pt3: BL	NR	4/M	2nd BL at 10 y	NHL-BFM90	CR at 18y
				Pt4: IL	NR	10/F	NR	NHL-BFM90 switched to CHOP because of progressive disease	Died 4.5 m after diagnosis
				Pt5: FL	NR	7/F	2nd DLBCL 13 m after diagnosis, third NHL (DLBCL) 8 y after diagnosis	NHL-BFM95	Current under treatment (NHL-BFM modified)
				Pt6; BL	NR	5/M	NR	NHL-BFM95	CR 6 y after diagnosis
				Pt7: DLBCL	NR	5/F	2nd malignancy: ALCL-T 3.5 y after diagnosis	NHL-BFM95 modified	Death of lung infection 8 y after diagnosis
				Pt8: DLBCL	NR	15/M	NR	NHL-BFM95 + Rituximab	CR 4 y after diagnosis
				Pt9: NHL	NR	10/F	NR	NHL-BFM95 modified	Death of lung infection 1 m after end of treatment
				Pt10: DLBCL	NR	9/F	2nd malignancy: T-ALL 3.5 y after diagnosis	B-NHL BFM04 modified + Rituximab	Death during treatment, 8 m after diagnosis of T-ALL
				Pt11: ALCL	NR	15/M	NR	NHL-BFM90	CR 6 y after diagnosis
Ataxia Telangiectasia	(38)	11	NS	Pt1: BL	NR	16/M	NR	B-NHL-BFM04 modified	Chronic lung disease, death of pulmonal infection 1.9 y after diagnosis
				Pt2: BL	NR	7/M	NR	B-NHL BFM04 modified + Rituximab-Window	CR 2.7 y after diagnosis
				Pt3: DLBCL	NR	6/M	NR	B-NHL BFM04 modified	CR 3.8 y after diagnosis
				Pt4: DLBCL	NR	9/F	NR	NHL-BFM90 modified	Death of early relapse 1 y after diagnosis
				Pt5: DLBCL	NR	5/M	2nd malignancy: DLBCL 10 y after diagnosis	NHL-BFM95 modified	CR
				Pt6: DLBCL	NR	11/M	2nd malignancy: DLBCL 3 y after diagnosis	NHL-BFM95 modified	Death with pulmonary failure
				Pt7: DLBCL	NR	7/F	NR	NHL BFM95 modified	CR 9.6 y after diagnosis
				Pt8: DLBCL	NR	10/F	NR	NHL-BFM95 modified	Death of therapy toxicity 4 m after diagnosis
				Pt9: DLBCL	NR	9/M	NR	NHL-BFM95 modified	CR 7.1 y after diagnosis
				Pt10: DLBCL	NR	9/F	NR	NHL-BFM95 modified	CR 7 y after diagnosis
				Pt11: HL	NR	12/M	NR	No chemotherapy	Death of progressive disease 1 m after diagnosis
NBS	(39)	10/26	Defective antitumor immunosurveillance	Pt1: NHL	NR	5/F	NR	NR	NR
				Pt2: NHL	NR	8/F	NR	NR	Death at 10 y
				Pt3: NHL	NR	7/F	NR	NR	Death at 7 y
				Pt4: NHL	NR	12/F	NR	NR	Death at 12 y
				Pt5: NHL	NR	7/F	NR	NR	Death at 10 y
				Pt6: NHL	NR	7/F	NR	NR	Death at 19 y
				Pt7: NHL	NR	7/F	NR	NR	Death at 7 y
				Pt8: NHL	NR	11/M	NR	NR	NR
				Pt9: NHL	NR	29/F	NR	NR	Death at 29 y
				Pt10: HL	NR	12/M	NR	NR	Death at 14 y
NBS^*^	(40)	11	Two cases possibly EBV associated	Pt1: BL	NR	9/M	Abdominal, splenomegaly (cervical tumor)	NR	Alive at 15 y
				Pt2: DLBCL	NR	24/M	Abdominal (inguinal LN)	NR	Died at 27 y
				Pt3: DLBCL	NR	11/F	Generalized (axillary LN)	NR	Died at 12 y
				Pt4: DLBCL	NR	6/F	Cervical LN	NR	Died at 7 y
				Pt5: DLBCL	NR	4/F	Cervical LN	NR	Died at 6 y
				Pt6: DLBCL	NR	15/F	Generalized (axillary LN)	NR	Died at 20 y
				Pt7: DLBCL	NR	11/M	Cervical LN	NR	Alive at 27 y
				Pt8: DLBCL	NR	4/M	Generalized cervical LN	NR	Died at 5 y
				Pt9: DLBCL	NR	8/F	Generalized (axillary LN)	NR	Died at 10 y
				Pt10: HL	NR	12/M	Cervical LN	NR	Died at 14 y
				Pt11: AILT-like	NR	8/F	Generalized cervical LN	NR	Died at 8 y
NBS	(41)	4	NS	Pt1: DLBCL	NR	15/F		NHL-BFM	CR, LFU +6 y
				Pt2: ALCL (B)	NR	6/F		NHL-BFM	Death of fungal sepsis after first course of therapy
				Pt3: DLBCL	NR	9/M		NHL-BFM	CR, LFU +2.5 y
				Pt4: IL	NR	10/F		NHL-BFM + RT	Initial nonresponse died after 5 m
NBS	(42)	1	Defective DNA repair	DLBCL	Homozygous 657del5	17/M	Bilateral cervical LN, malaise, headache, epistaxis, symptoms of URI, fever, night sweats, and loss of weight, followed by the development of protruding tissue mass in the epigastrium	Modified CHOP + Rituximab	CR, 3 y in CR on LFU
NBS^*^	(43)	1	EBV associated	HL	Homozygous 657del5	5/F	Fever lasting for 2 m and mediastinal adenopathy	COPP/ABV	CR, 2 y in CR on LFU
NBS^*^	(44)	12/57	Chronic antigenic stimulation	NHL	NR	NR	NR	NR	15 out of total 22 patients died, but 7 were clinically stable after early diagnosis and successful treatment
		2/57	Chronic antigenic stimulation	HL	NR	NR	NR	NR	
NBS	(45)	30/149	NS	NHL	NR	NR	NR	NR	44% of all patients in CR, 54% dead due to disease progression
		7/149		HL	NR	NR	NR	NR	
Dyskeratosis Congenita	(46)	1	Genetic insatiability	HL	NR	30/M	NR	Radiation-chemotherapy	Died after 25 y (gastric adenocarcinoma)
Ataxia Telangiectasia	(3)	1/10	NS	1 NHL	NR	NR	NR	NR	NR
Ataxia Telangiectasia	(41)	2	NS	Pt1: DLBCL	NR	9/F		NHL-BFM	Relapse after 10 m, died after 1 y of diagnosis
			NS	Pt2: DLBCL	NR	12/M		NHL-BFM	CR, LFU +1 y
PNP deficiency	(41)	1	NS	ALCL (B)	NR	2/F		dosages reduced	BMT (haploident.) in CR, BMT-related death
Schimke Immuno-Osseous dysplasia	(47)	1	NR	NHL	SMARCAL1 missense mutation (R561H)	8/M	Colicky abdominal pain and vomiting. Palpation of the abdomen revealed a hard mass in right upper abdomen. Intussusception secondary to NHL	Vincristine, cyclophosphamide, adriamycin and intrathecal methotrexate using half of their usual doses	Died due to septicemia following chemotherapy
Schimke Immuno-Osseous dysplasia	(48)	3/71	NS	NHL	SMARCAL mutation	NR	NR	NR	NR
Schimke Immuno-Osseous dysplasia^*^	(49)	1	EBV associated	BCL	Homozygous mutation of the SMARCAL1 gene (1146–1147delAA þ IVS6 þ 2delGT)	5/M	Fever, mild cough	Chemotherapy	Died 1 m later due to multiorgan failure
CHARGE association with Hyper-IgM	(50)	1	Chronic antigenic stimulation	MZL	NR	5/F	Suspected purulent bilateral conjunctivitis: Unresponsive On microscopy: salmon-colored, nodular lesions	Topical IFN-[alpha] 3 times a day 300,000 U/drop.	Resolved. No lesions at 1 y FU
**PREDOMINANTLY ANTIBODY DEFICIENCIES**									
Severe Ig deficiency	(41)	1	NS	DLBCL	NR	14/F		VBL for palliation only	Died after 3 m
IgG1, −3 and −4 deficiency	(41)	1	NS	BL	NR	6/F		NHL-BFM	CR, LFU +5 y
Selective IgA deficiency	(41)	1	NS	Burkitt-like	NR	2/M		NHL-BFM	CR, LFU +0.5 y
IgG-4 and IgM deficiency	(41)	1	NS	BL	NR	5/F		B-NHL therapy per protocol full dosage	CR, LFU +4.5 y
IgA, IgG2, and −4 deficiency	(41)	1	NS	BL	NR	11/M		NHL-BFM	Reached CR, died of sepsis after second therapy course
APDS	(51)	2/8	NS	Pt1: DLBCL	GOF mutation in the PIK3CD gene: E1021K (c.3061G > A)	8/M	2nd DLBCL at 19 y	Initially UKCCSG 9002 protocol, 2nd malignancy: CHOP + Rituximab	Died from large bowel perforation and bleeding 12 days after the third course of chemotherapy.
				Pt2: HL	Heterozygous GOF mutation in the PIK3CD gene: E1021K	11/M	Cervical LN enlargement	Chemotherapy and RT	CR, alive FU of more than 10 y
APDS	(52)	6/53	NS	2 DLBCL, 1 HL, 1 NMZL,1 Hodgkin-like	E1021K mutation	1.5–27/NR	NR	NR	3 died
APDS	(53)	1/17	NS	MZL	E1021K mutation	NR	NR	NR	NR
Selective IgA deficiency	(54)	1/386	NS	NHL	NR	38/M	NR	NR	NR
CVID	(55)	1	NS	DHL	NR	47/M	Abd pain, numbness, and weakness in legs.	RT, cyclophosphamide, vincristine, procarbazine, prednisone	FU 3.5 y: CR
CVID	(56)	1	NS	EZMZL		16/F	Cough, wheezing	Prednisolone	Alive
CVID	(57)	1	NS	EZMZL		44/F		Chlorambucil and prednisone	Recurrence after 6m
CVID	(58)	54	EBV possibly	BCL	NR	NR	NR	NR	NR
CVID^*^	(59)	3/220	EBV associated	BCL	NR	NR	NR	NR	NR
CVID	(60)	7	NS	BCL	NR	46^**^/F	NR	NR	NR
		9	NS	BCL	NR	42^**^/M	NR	NR	NR
CVID^*^	(61)	1	EBV or ITK mutation	HL	NR	25/F	NR	Rituximab and brentuximab vedotin.	CR
CVID	(62)	3	NS	Pt1: BCL	NR	2/M	Generalized LN	NR	Died at 23y
				Pt2: BCL	NR	61/F	NR	NR	NR
				Pt3: BCL	NR	66/F	NR	NR	NR
CVID	(63)	22/248	NS	Pt1: NHL	NR	13/F	NR	Chemotherapy (CHOP)	Died, age 13
				Pt2: NHL	NR	31/F	NR	Surgery	Alive 1 y later
				Pt3: NHL	NR	41/M	NR	Chemotherapy, HSCT	Died, age 41
				Pt4: NHL	NR	44/F	NR	Surgery	Unknown, alive 8 y later
				Pt5: NHL	NR	45/M	NR	None	Alive, 1 y later
				Pt6: NHL	NR	46/F	NR	Chemotherapy (Type?)	Died
				Pt7: NHL	NR	48/M	NR	None	Died, age 48
				Pt8: NHL	NR	52/F	NR	Chemotherapy (CHOP)	Died, age 56
				Pt9: NHL	NR	52/F	NR	Radiation	Alive, 2 y later
				Pt10: NHL	NR	54/F	NR	Surgery, surgery, chemotherapy	Alive at age 68
				Pt11: NHL	NR	54/F	NR	Surgery	Died other causes, 15 y later, age 69
				Pt12: NHL	NR	56/F	NR	M-BACOD, CHOP, RT, CP	Alive, 12 y later
				Pt13: NHL	NR	63/M	NR	CHOP, M-BACOD, M2	Died, age 65
				Pt14: NHL	NR	67/F	NR	Chemotherapy (C-MOPP)	Died, age 68
				Pt15: NHL	NR	67/F	NR	Chemotherapy (CHOP)	Alive, stable 8 y later
				Pt16: NHL	NR	71/F	NR	Chemotherapy (C-MOPP)	Died, age 72
				Pt17: NHL	NR	72/F	NR	Alpha interferon	Died, age 73
				Pt18: NHL	NR	75/F	NR	Radiation	Alive, stable, 2 y later
				Pt19: NHL	NR	77/M	NR	Chemotherapy (Type?)	Died, age 77
				Pt20, Pt 21, Pt 22: HL	NR	8/M Rest NR	NR	Splenectomy and chemotherapy in Pt20	Pt20 relapsed after 12 years
CVID	(64)	5/5	NS	Pt1: EZMZL	NR	56/F	Parotid, 2nd Breast, 3rd DLBCL in sternum after 11 y	Radiation, excision, CHOP, rituximab	Recurrence
				Pt2: EZMZL	NR	35/F	Lung	None	Well
				Pt3: EZMZL	NR	31/F	Parotid	Excision, radiation	Recurrence
				Pt4: EZMZL	NR	42/M	Lung involvement	CVP and Rituximab 2, CHOP	Recurrence
				Pt5: EZMZL	NR	35/F	Lung	R-CHOP	CR
CVID	(65, 66)	9/117	Unknown	Pt1: DLBCL	NR	12/F	Liver, Spleen	CHOP	Died at 13 y
				Pt2: DMSL	NR	54/F	Right Inguinal Node	CHOP	Died at 69 due to other causes
				Pt3: DMSL	NR	50/F	Pelvis,	CHOP	Died at 56 y
				Pt4: DLBCL	NR	48/F	Proximal jejunum	Surgery	Alive, 8 y later
				Pt5: Follicular	NR	54/F	Parotid, 1 y later FL in axilla	Surgery for both	Alive, after 3 y
				Pt6: diffuse small cleaved cell	NR	56/F	Pelvic nodes	M-BACOD, CHOP, RT, CP	Alive
				Pt7: DLBCL	NR	57/F	Supraclavicular area, abdomen	C-MOPP	Died at age 68
				Pt8: DLBCL	NR	65/F	Lymph nodes, lungs	C-MOPP	Died at age 72
				Pt9: DLBCL	NR	70/F	Cervical lymph nodes	Alpha-Interferon	Alive
CVID	(54)	5/176	NS	Pt1: NHL	NR	52/F	NR	NR	NR
				Pt2: NHL	NR	59/F	NR	NR	NR
				Pt3: NHL	NR	76/F	NR	NR	NR
				Pt4: HL	NR	49/F	NR	NR	NR
CVID	(67)	1	Defective antitumor immunosurveillance	DHL	NR	44/F	Abd. pain, slight diarrhea, weight loss. Palpable mass in right abdomen	Resection and irradiation	4-y F/U: CR
CVID	(68)	1	Chronic antigenic stimulation	IL	NR	75/W	Weight loss, numbness and tingling in extremities	Surgical resection	Death post-op due to septicemia
CVID	(69)	1	Defective antitumor immunosurveillance	MZL	NR	39/M	Periumbilical pain, severe diarrhea, weight loss	NR	NR
CVID	(70)	4/224	NS	NHL	NR	NR	NR	Chemotherapy + Rituximab + transplant	NR
CVID	(71)	5/247	NS	Jejunal, 1 histiocytic, 1 HL, 2 BCL	NR	NR	NR	NR	4 died, HL responded to RT
CVID^*^	(72)	1	NS	EMZL	NR	41/F	Malaise, dyspnea and productive cough	IVIG, chlorambucil and prednisone	
CVID	(73)	10/334	NS	NHL	NR	NR	NR	NR	5 died.
CVID	(3)	10/416	NS	1 DLBCL, 2 FL,1 BL, 1 SLL,1 EMZL, 3 NHL,1 WM	NR	NR	NR	NR	NR
CVID	(74)	23	NR	NHL	NR	NR/28F, 11M	NR		11 died of lymphoma, 12 alive
		3	NR	DLBCL	NR				2 died of lymphoma, 1 also had severe lung disease, 1 alive
		4	NR	HL	NR				2 died of lymphoma, 2 alive
		5	NR	EMZL	NR			3 no treatment, 2 chemotherapy	All alive
		1	NR	MZL	NR				No treatment given, alive
		1	NR	Diffuse poorly differentiated	NR				Died of lymphoma
		1	EBV associated	T -cell rich BCL	NR				Died of lymphoma
Selective IgA deficiency	(75)	1	Chronic antigenic stimulation	PCMZL, MZL	MZL: monoclonal amplification of 98 base pairs in FR3A region of the IGH gene	43/F	Asymptomatic subcutaneous nodules in upper extremity. Enlarged axillary, mediastinal LN	RituximabRadiotherapy and local excision	CR followed by repetitive relapses
**DISEASES OF IMMUNE DYSREGULATION**									
ALPS^*^	(76)	1	EBV associated	FL, DLBCL, HL	c.784A>T mutation in TNFRSF6,	33/M	Lymphadenopathy	R-CHOP	CR
XLP1	(77)	3	Defective antitumor immunosurveillance	Pt1: BL	SH2D1A p.E17D c.51 G > C	6/M	Acute abd. obstruction on presentation, night sweats, weight loss.	NHL-BFM95, full dosage, + Rituximab	CR
				Pt2: DLBCL	SH2D1A p.W64C c.192G > T	14/M	Painless mass of about 2.0 × 2.5 cm in size on the front right-side chest wall	B-NHL-M2004, full dosage + Rituximab	Died at the age of 19
				Pt3: DLBCL	SH2D1A c.53insA > T	6/M	Testicular DLBCL	NHL-BFM90 + Rituximab	Died at 8.3 y
XLP1^*^	(78)	1	NS	BL	exon 2 of the SH2D1A gene at position 146 (c146insG	4/M	Lymphadenopathy, HS, cerebellar tumor	ETO, DXM, cyclophosphamide	PR died of ICH
XLP^*^	(79)	2	EBV associated	Pt1: BL	NR	1.6/M	Palpable abd. mass Malaise, poor appetite, failure to thrive.	Protocol LMB-84	Died of hemorrhage 6 m after stopping therapy.
				Pt2: BL	t(8;14) (q24;q32)	2/M	Abd. Mass, malaise and failure to thrive.	Protocol LMB-84	Relapse after 17 m. Died.
ITK deficiency^*^	(80)	1	EBV associated	HL	48, X,X,+2,der(10)add(10)(p13) add(10)(q34),del(11)(q22q23), + 12,-13, + 1B2mar	5/F	Cervical and inguinal lymphadenopathy	IFS, vinorelbine, etoposide, cytarabine, gemcitabine) RT, HSCT	CR
ITK deficiency^*^	(81)	2	EBV associated	Pt1: NHL	NS	6/F	Cervical and occipital Lymphadenopathy	CHOP and anti CD20 Ab	Died of infection
				P2: HL	NS	2.5/M	NR	Chemotherapy	NR
ITK deficiency^*^	(82)	1	EBV associated	HL	NS		Lung and Kidney	HSCT, Rituximab, Fludarabine, Melphalan, ATG	CR
ITK deficiency^*^	(83)	4	EBV associated	Pt1: HL	IKT gene: 1764C>G: YS886	4/F	LN	NR	Died at age 6
				Pt2: HL	IKT gene: 1764C>G: YS886	5/M	LN	Induction Therapy	PreHSCT, age 10
				Pt3: HL	IKT gene: 1764C>G: YS886	3/M	LN	HSCT	Alive
				Pt4: DLBCL	IKT gene: 1497delT: D500T, F501L, MS03X	6/F	Lungs	HSCT	Died from GVHD in 6 m
ITK deficiency^*^	(84)	3	EBV associated	Pt1: HL	ITK gene: single homozygous mutation c. 1764 C->G which causes a premature stop-codon Y588X	5/F	Lymphadenopathy and Hepatosplenomegaly	Rituximab, VP-16, vinorelbine, GMC	Died of respiratory failure
				Pt2: HL		5/M	Lymphadenopathy	Chemotherapy	CR
				Pt3: HL		3/M	Lymphadenopathy and HS	HSCT with fludarabine, melphalan, ATG, rituximab	Alive
ITK deficiency^*^	(85)	2	EBV associated	Pt1: HL	Homozygous mutation in ITK on ch 5q31–5q32.	6/F	Generalized LN, HS, nasal, concha tumor	Prednisone, procarbazine, vincristine, cyclophosphamide, ADM + rituximab	Died of respiratory failure at 10
				Pt2: HL	homozygous mutation in ITK 5q31–5q32.	1/F	Retroperitoneal and abd. LN, HS	GNC, steroid, rituximab, HSCT	Died of ischemic brain injury
CD70 deficiency^*^	(86)	1	EBV associated	HL		3/M	Recurrent fever, lymphadenopathy, HS	Initially chemotherapy and radiotherapy Rituximab at 4 HSCT at 10	CR after chemo/radiotherapy No relapse of HL, however recurrent lymphoproliferative episodes. Well since HSCT.
CD70 deficiency^*^	(87)	3	EBV associated	Pt1: HL	Homozygous c.250delT resulting in p.S84Pfs27X	17/M	Peptic ulcer, gastritis, splenomegaly and lymphadenopathy	doxorubicin, bleomycin, vinblastine, and dacarbazine	CR, F/U at 29 y: Stable
				Pt2: HL	Homozygous c.555_557delCTT resulting in p.F186del	2/M	Diffuse cervical lymphadenopathy	Initially OPPA and COPP ABVD + radiotherapy after relapse	CR after ABVD/radiotherapy F/U at 16: CR
				Pt3: HL	Homozygous c.555_557delCTT resulting in p.F186del	3/M	Chronic cervical lymphadenopathy	ABVD gemcitabine, vinorelbine, and brentuximab were followed by auto-HSCT	Relapse after ABVD, CR after HSCT
CD27 deficiency^*^	(88)	5	EBV associated	Pt1: DLBCL	Homozygous c.G158A, p.C53Y	2/F	NR	None	Died at 2 y
				Pt2: DLBCL	Homozygous c.G158A, p.C53Y	22/F	NR	CHOP	Died at 22
				Pt3: HL	Compound c.G24A, p.W8X; c.C319T p.R107C	15/F	Persistent cervical lymphadenopathy	EuroNet-PHL-C1	Alive
				Pt4: HL	Homozygous c.G287A, p.C96Y	?/F	NR	None	Died at 20 y
				Pt5: HL	Homozygous c.G287A, p.C96Y	8/M	NR	ABVD, radiotherapy	CR
XMEN^*^ (MAGT1 deficiency)	(89, 90)	2	EBV associated	1. BL	g.43183delC	7/M	2nd malignancy at 14	NR	Alive at 16 y
				2. HL	g.46604G>T	17/NR	2nd malignancy at 22	HSCT	Died at 23 y of HSCT complications
XMEN	(91)	1	EBV associated (suspected)	DLBCL	c.712C > T, p.R238X	57/M	Lymphadenopathy, HS and B-symptoms	RCHOP, RGCVP	Died at 58 y from DLBCL
XMEN^*^	(92)	1	EBV associated	HL	c.555dup, p. Tyr186Ilefs^*^2	15/M	Influenza-like symptoms	COPP and ABVD	Alive at 17 y
CTPS1 deficiency^*^	(93)	2	EBV associated	Pt1: NHL	NR	6/F	NR		Died at 6 y
				Pt2: NHL	NR	1/M	NR	HSCT	Alive at 2 y
Hypergammaglobulinemia	(41)	1	NS	DLBCL	NR	1/F		No therapy received	Died of disease progression after 6 m
IL-10R deficiency	(94)	5	Defective antitumor immunosurveillance	Pt1: DLBCL	IL-10RB gene: homozygous missense in exon 2 (p.Y59C)	5/M	NR	COP, COPADM 3 2, CYM 3 2	Died of disease progression
				Pt2: DLBCL	IL-10RB gene: heterozygous composite frameshift mutation in exon 7 (F269fsX275) + missense mutation in exon 5 (p.W204C)	5/M	NR	COP, R-COPADM	Died during treatment
				Pt3: DLBCL	homozygous nonsense in exon 3 of IL-10RB (p. E141X)	6/M	NR	COP, R-COPADM, R-CYM, HSCT	Remission, alive (+12 m)
				Pt4: DLBCL	homozygous del (g.11930-17413 del) in IL-10RB.	5/M	NR	COP, COPADM, R-CYM, R-ICE	Remission, alive (+18 m)
				Pt5: DLBCL	Homozygous c.368-10 C.G in intron 3 of IL-10RA.	6/M	NR	Up-front R prephase	Remission, alive
**DEFECTS IN INTRINSIC AND INNATE IMMUNITY**									
Whim Syndrome^*^	(95)	1	EBV associated	BCL	NR	30/M	Red dermal facial nodules Axillary mass Axillary, hilar, Inguinal LN	6 cycles CHOP + G-CSF before each cycle	Resolved after 6 cycles.
Whim Syndrome^*^	(96)	1	EBV associated	BCL	NR	26/F	Fatal EBV+ B cell Lymphoma in the intestine and other organs.	CHOP	No response. Died of perforation and hemorrhage

*Abd, (abdominal); ABVD, (doxorubicin [Adriamycin], bleomycin, vinblastine, and dacarbazine); AILT, (angioimmunoblastic T cell lymphoma); APO, (doxorubicin, vincristine, 6-MP, and prednisone); APDS, (activated phosphoinositide 3-kinase d syndrome); BCL, (B cell lymphoma); BL, (Burkitt lymphoma); ALPS, (Autoimmune lymphoproliferative syndrome); COP, (cyclophosphamide, vincristine, prednisone); COPADM, (cyclophosphamide, vincristine, prednisone, doxorubicin, methotrexate); CHOP, (cyclophosphamide, adriamycin, vincristine, prednisolone); COPP, (Cyclophosphamide, Oncovin, Procarbazine, Prednisone); CP, (cis-platinum); C-MOPP, (cyclophosphamide, oncovin, procarbazine, prednisone); CYM, (cytarabine, methotrexate); DHL, (diffuse histiocytic lymphoma); EMZL, (extra nodal marginal zone lymphoma); F, (female); G-CSF, (Granulocyte colony-stimulating factor); HLH, (hemophagocytic lymphohistiocytosis); ICE, (ifosfamide, carboplatin, etoposide); IL, (immunoblastic lymphoma); INHL, (immunoblastic NHL); LFU, (last follow up); LN, (lymph node); LPD, (lymphoproliferative Disease); M, (male); m, (months); M-BACOD, (methotrexate, doxorubicin, cyclophosphamide, vincristine, dexamethasone); ML, (Malignant Lymphoma); M2, (vincristine, cyclophosphamide); NHL-BFM, (Non-Hodgkin Lymphoma-Berlin-Frankfurt-Münster); NHL, (Non-Hodgkin Lymphoma); NMZL, (nodal marginal zone lymphoma); LL, (lymphoplasmacytic lymphoma); n, (number of patients); OPPA, (Oncovin, procarbazine, prednisone, and doxorubicin); PDC, (plasmacytoid dendritic cells); pt, (patient); PCMZL, (primary cutaneous marginal zone lymphoma); PE, (Physical Examination); PTCL, (peripheral T cell lymphoma); RT, (radiation therapy); R, (Rituximab); WM, waldenstrom macroglobulinemia; XLP1, (X-linked lymphoproliferative disease type I); y, (years)*.

* *EBV positive*,

** *median age*.

## B Cell Lymphomas in PIDDs

A thorough search of the literature using Medline database (via PubMed; Figure 1) identified 86 studies reporting B cell lymphoma in PIDD patients. Table 1 gives details of the 456 patients identified from literature plus two unpublished cases from our center (Mayo Clinic, Rochester). The types of B cell lymphoma were unspecified non-Hodgkin Lymphoma (NHL) (37%, *n* = 171), diffuse large B cell lymphoma (DLBCL) (15%, *n* = 68), Hodgkin lymphoma (HL) (13%, *n* = 59), marginal zone lymphoma (MZL) including extranodal and intranodal MZL (5%, *n* = 23), Burkitt lymphoma (BL) (4%, *n* = 17) and diffuse histiocytic lymphoma (DHL) (0.4%, *n* = 2). Unlike T cell lymphomas where most of cases were reported in males, gender distribution was similar in males (29%, *n* = 130) and females (34%, *n* = 157), it was not specified (NS) in 37% (*n* = 169). The age of onset/diagnosis of lymphoma ranged from 7 months to 76 years (median age: 12 years). EBV association was seen in 25% (*n* = 113) of the patients. The majority of patients received combination chemotherapy as a standard treatment. While allogeneic hematopoietic cell transplantation (HCT) was not performed in many of these cases, it appeared to be successful in achieving a complete response (CR) in some cases. Serious infectious complications and death were frequently associated with chemotherapy treatment. Individual groups of PIDDs are examined in further detail based on the IUIS classification.

### IUIS: Immunodeficiencies Affecting Cellular and Humoral Immunity

There were 12 studies which included 13 patients with B cell lymphoma. Adenosine deaminase 1 (ADA1) deficiency was most common immunodeficiency disease in this category (19–24). The other 7 cases include patients with an underlying diagnosis of Coronin 1A and DNA Ligase IV deficiencies, Artemis-SCID, RAG1, and ZAP70 defects (13–18, 24, 107). Of these 13 cases, 69% (*n* = 9) patients were females, 15% (*n* = 2) were males and 15% (*n* = 2) were NS. DLBCL (62%, *n* = 8) was the most common type of B cell neoplasm identified followed by unspecified B cell lymphoma (15%, *n* = 2). The median age at presentation was 1.5 years (range 0.9–14 years) and all patients were EBV-positive. The most common clinical presentation was lymphadenopathy and high fevers. The underlying etiology for the development of lymphoma appeared to be DNA repair defects and EBV association. All patients were treated with some form of combination chemotherapy, most commonly, rituximab, cyclophosphamide, doxorubicin, vincristine, and prednisolone (R-CHOP). Of these 7 cases, only 2 patients survived and were in complete remission at follow-up. One patient who developed DLBCL in the setting of RAG1 deficiency had a partial response with rituximab, which was consolidated by HCT from an HLA-matched unrelated donor. Though there is no long-term follow-up data in this report, the patient had no evidence of disease at 3 years follow-up (16).

#### Adenosine Deaminase 1 (ADA1) Deficiency

B cell lymphoma with ADA1 deficiency was seen in six patients, three of whom had EBV association (19–24). Only one patient was reported to be in complete remission 20 months after diagnosis, others died despite treatment.

### IUIS: Combined Immunodeficiency Disorders With Associated or Syndromic Features

In this category, 27 studies plus two unpublished cases reported B cell lymphomas in 191 patients with combined immunodeficiency disorders with associated or syndromic features. Of these, 41% (*n* = 78) cases were associated with EBV infection, and the lymphoma was diagnosed at median age of 10 years. In this cohort there appeared to be a preponderance of males at 38% (*n* = 73) with females accounting for 32% (*n* = 62) and 30% (*n* = 56). Some of the more common genetic disorders are discussed further.

#### Ataxia Telangiectasia (AT)

Over one-third (35%, *n* = 67) of the reported cases of lymphoma in this IUIS category were in the setting of ataxia telangiectasia, which is an autosomal recessive disorder with immunodeficiency, DNA repair defects and neurological complications(3, 25–28, 38, 41). Lymphomas were diagnosed across all ages of patients typically manifesting before 10 years of age with the oldest cases being reported at 16 years of age. Original reports reported the histology as DLBCL or unspecified NHL in 75% (*n* = 50), HL in 21% (*n* = 14), BL in 3% (*n* = 2), and MZL in 1% (*n* = 1) of cases. The most common presenting symptoms included extensive lymphadenopathy. A French study reported a crude incidence of cancer of 24.7% in AT with B cell lymphoid malignancies representing the majority of cases. The pathological diagnosis of lymphoma in these patients can be challenging, and it is relevant to note that three of the four T-cell NHLs, based on pathology, were reclassified as B-cell NHLs after centralized pathology review. The median overall survival (OS) decreased from 24 to 15 years in AT patients with malignant disease. In fact, the common causes of mortality in this group were either cancer (47%) or infectious complications (34%). There was a trend toward increased survival if there was an excellent response to chemotherapy. Though the overall prognosis in AT remains relatively poor, a subset of these of patients might benefit from treatment of the malignancy with an improved survival (25).

#### Wiskott -Aldrich Syndrome (WAS)

There were 6 studies identified, which reported B cell lymphoma in 6 patients (median age 14 years) with WAS, which is an X-linked PIDD (29–34). DLBCL (33%, *n* = 2) was the most common type of B cell lymphoma in this group, followed by BL, HL, unspecified NHL and immunoblastic B cell lymphoma. As with many of the other PIDDs, EBV association was present in all these types. It is relevant to note that only 33% patients (*n* = 2, HL and Burkitt lymphoma) had complete remission on follow-up, while the others died of disease progression or its complications. In a multi-center cohort of X-linked thrombocytopenia (XLT) patients, certain *WAS* genetic variants associated with XLT, a milder form of WAS, predisposed to a higher incidence of developing lymphoma suggesting that these patients would benefit from treatment with HCT (108).

#### DiGeorge Syndrome (DGS)

In this literature review, DGS did not appear to be associated with a high incidence of lymphomas, with only one reported case in a 10-month female who developed immunoblastic B cell NHL(35). Similar to other PIDDs this case was also associated with EBV infection. The patient manifested multi-system complications with hemiparesis due to a cerebral mass, mediastinal lymphadenopathy, liver and kidney involvement, and succumbed to the disease prior to initiation of therapy.

#### Cartilage Hair Hypoplasia (CHH)

CHH is a syndromic PIDD due to genetic defects in the *RMRP* gene and manifests with variable degree of immunodeficiency. While many patients who manifest with severe cellular immunodeficiency early in life may receive an HCT, patients with initially milder immunodeficiency may develop complications later in life, including malignancy. We identified 10 reported cases, with a median age of 29 years, equally divided between males and females. Majority of these cases were NHL (90%, *n* = 9) while the remaining (10%, *n* = 1) were HL. Interestingly, none of these were associated with EBV (36). This is in contrast to the 2 cases of adult CHH (male patients) in our center (unpublished, Mayo Clinic, Rochester), who were both EBV-positive and diagnosed in their 3rd to 4th decade of life. One of these patients was diagnosed with DLBCL after an incidental work-up of a lung nodule, while the other patient had MZL with recurrent fevers and night sweats. The patient with DLBCL was treated with R-CHOP and had CR, while the patients with MZL had recurrent disease, and was treated with rituximab. Therefore, like other PIDDs, CHH may also present with EBV-associated lymphomas, and it remains unclear if the EBV diagnosis could have been missed in other cases reported in the literature.

#### Nijmegen Breakage Syndrome (NBS)

In this review strategy, 9 studies with 97 cases of lymphomas in patients with NBS were identified (37–45). NBS is a syndromic DNA repair defect, and in these published reports, 76% (*n* = 74) of patients presented with unclassified NHL and DLBCL. Most cases (82%, *n* = 80) were EBV-negative. The underlying DNA repair defect puts these patients at high risk, unless they receive HCT early after diagnosis.

#### Dyskeratosis Congenita (DC)

Among the short telomere syndromes, while one would postulate high-risk of malignancy due to premature cellular senescence, this literature search only yielded a single case of an adult DC patient with HL not associated with EBV (46). The treatment strategy included combination chemotherapy and radiation, which likely was not appropriate in the context of the patient's underlying genetic defect, and presumed radiosensitivity, as the patient subsequently developed a gastric carcinoma and died of the disease. Short telomere syndromes are known to be associated with radiosensitivity, and therefore, radiation therapy is probably not a recommended treatment modality in this group, as well as in the patients with DNA repair defects.

#### Schimke Immuno-Osseous dysplasia

We identified 3 studies, with 5 cases of B cell lymphoma (47–49). EBV association was seen in a single case (20%, *n* = 1). Outcome data was given for two patients, both of whom died with complications soon after starting chemotherapy.

#### CHARGE Association With Hyper IgM Syndrome

We identified one report of a 5-year-old girl, with nonclassical CHARGE association and elevated IgM levels who developed bilateral extranodal (ocular) MZL. She was treated with topical interferon alpha with subsequent complete resolution of disease (50).

### IUIS: Predominantly Antibody Deficiencies

In this literature review, we identified 27 studies, which reported B cell lymphomas in 208 patients with predominantly antibody deficiencies. The median age at presentation was 46 years (range: 2–76 years) with a gender distribution of 34% (*n* = 71) female, 13% (*n* = 27) male, and 53% (*n* = 110) NS.

#### Common Variable Immune Deficiency (CVID)

Twenty-two studies of 192 CVID patients with B-cell lymphoma are presented in Table 1 (3, 54–74). Common lymphomas included unclassified NHL (32%, *n* = 62), MALT lymphoma (EMZL) (7%, *n* = 14), DLBCL (5%, *n* = 9), and HL (4%, *n* = 8). Of these, 31% (*n* = 60) cases appeared to be associated with EBV infection. Possible mechanisms of lymphomagenesis in these different cohorts included chronic antigenic stimulation and defective immune surveillance. The treatments of choice in these patients included surgical resection and/or radiotherapy with chemotherapy. Common Variable Immunodeficiency (CVID) is the most common adult humoral immunodeficiency, both in US and European studies. There are several reports documenting an increased risk of lymphoma in these patients. In a large cohort of 176 patients with CVID, an increased incidence of lymphoma (obs = 4; SIR = 12.1; 95% CI = 3.3–31.0) was noted (54). In an ESID (European Society for Immunodeficiencies) registry study of 2,212 CVID patients, a subset (*n* = 902) analysis identified 3% of patients with lymphoma (106). However, in another study from a single center, which followed 473 CVID patients over four decades, the incidence of lymphoma was higher at 8.2% (74).

#### Selective IgA Deficiency

Three studies reported three cases of selective IgA deficiency with B cell lymphoma; an adult patient with apparent selective IgA deficiency who presented with a primary cutaneous MZL, a 38-year-old male with unclassified NHL and a 2-year-old boy with Burkitt-like lymphoma (41, 54, 75). It is relevant to note that a combined Danish and Swedish study of 386 patients with IgA deficiency did not show an increased risk for cancer (standardized incidence ratio = 1.0) (54).

#### Activated Phosphoinositide 3-Kinase D Syndrome (APDS)

Nine patients with gain of function (GOF) mutation in PIK3CD gene (APDS) developed B cell lymphoma in the three studies identified in this review (51–53). DLBCL was the most common type (33%, *n* = 3). An EBV association was not seen in these particular cases.

#### Other Ig Deficiencies

Seidemann et al. reported four cases of B cell lymphoma in different Ig subclass deficiencies (excluding selective IgA deficiency) (41). Seventy-Five Percent (*n* = 3) had Burkitt lymphoma (BL). Two died during treatment with chemotherapy, two were alive and in complete remission at last follow up.

### IUS: Diseases of Immune Dysregulation

In this category, 42 cases from 20 studies were identified, and of these, 76% (*n* = 32) had an association with EBV (41, 76–94). The median age of the reported cases was 5 years (range: 1–33 years), 62% (*n* = 26) were male, 31% (*n* = 13) female and 7% (*n* = 3) were NS. HL (48%, *n* = 20) was the most common subtype in these disorders of immune dysregulation, followed by DLBCL (29%, *n* = 12), BL (12%, *n* = 5), unspecified NHL (7%, *n* = 3) and composite lymphoma—FL, DLBCL, HL (2%). Specific examples are discussed further.

#### X-linked Lymphoproliferative Syndrome Type 1 (XLP-1)

In patients with X-linked lymphoproliferative syndrome type 1 due to genetic defects in *SH2D1A*, there were 3 studies with six patients who developed B cell lymphoma [4 BL, 2 DLBCL (77–79)]. The median age at diagnosis of lymphoma was 5 years (range: 1–14 years), and of these cases, 50% (*n* = 3) were EBV-associated and 50% (*n* = 3) had no apparent EBV association. Since XLP1 is associated with a defective immune response to EBV related to impaired cellular cytotoxicity it is not unexpected that there would be impaired immune surveillance in these patients (109). The two patients who had a DLBCL not related to EBV presented with a testicular mass and right chest mass, respectively. In the cases with the BL, clinical presentation involved lymphadenopathy and palpable abdominal mass/obstruction or cerebellar tumor. With the exception of a single case, all patients died within 5 years of diagnosis regardless of treatment modality used.

#### Interleukin-2-Inducible T-Cell Kinase (ITK) deficiency

Patients with ITK deficiency have an intrinsic susceptibility to EBV. In six studies, 13 patients with ITK deficiency (Table 1) who developed B cell lymphoma were identified, and all were associated with EBV (80–85). The most common was HL (84%, *n* = 11) followed by NHL (7%, *n* = 1) and DLBCL (7%, *n* = 1). The median age at presentation was 5 years (range: 1–6) with 54% (*n* = 7) female and 46% (*n* = 6) male, presenting clinically with lymphadenopathy and hepatomegaly. The treatment of choice was chemotherapy and HCT.

#### Autoimmune Lymphoproliferative Syndrome (ALPS)

While there are several genetic defects associated with ALPS or ALPS-like disease, the most frequent genetic defect associated with a classic ALPS-like phenotype is heterozygous germline pathogenic variants in the *FAS* gene. In Table 1, there was a single report an EBV-associated composite lymphoma (FL, DLBCL, HL) in an adult male with ALPS, which was treated with R-CHOP resulting in CR (76).

#### IL10-R Deficiency

Monogenic inflammatory bowel disease (IBD) can be associated with complex presentations, and patients with genetic defects in the IL-10 receptor (*IL10RA* and *IL10RB*) have particularly severe disease with additional complications. A single case report (110) and a case series has reported DLBCL in 5 patients with IL10R deficiency (94). There was no EBV association noted in any of these cases, and the median age of developing lymphoma was 5 years (range: 5–6 years). Interestingly, all patients in these series were male. Chemotherapy with COP (cyclophosphamide, vincristine, prednisone), COPADM (cyclophosphamide, vincristine, prednisone, doxorubicin, methotrexate), CYM (cytarabine, methotrexate), and ICE (ifosfamide, carboplatin, etoposide) was used with variable success. Among the five patients, two died due to disease progression during treatment, while three who received HCT remained alive and in remission on follow-up. It is evident that in this context, HCT is not only curative but may very well prevent the occurrence or recurrence of lymphoma (111).

#### CD70 Deficiency

B cell lymphoma was reported in four male patients under the age of 20 with CD70 deficiency across two studies (86, 87). All patients presented with HL, associated with EBV infection and were managed with chemotherapy and radiotherapy. Two patients underwent HCT and complete remission was achieved in all cases.

#### CD27 Deficiency

We identified one study reporting five cases (mostly females) with CD27 deficiency who developed B cell lymphoma (88). Three patients were diagnosed with HL, and two with DLBCL. All were related to EBV infection. Only two patients with HL treated with the EuroNet-PHL-C1 (EuroNet-Pediatric Hodgkin's Lymphoma Group-C1) protocol and ABVD (doxorubicin, bleomycin, vinblastine, dacarbazine) plus radiotherapy respectively were alive and in complete remission.

#### XMEN (MAGT1) Deficiency

Four studies with four patients were reported to develop B cell lymphoma in the context of MAGT1 deficiency (89–92). All were males with EBV-associated lymphomas. The ages ranged from 7 to 57 years, and the distribution of B cell lymphomas included 2 HL, 1 BL, and 1 DLBCL.

#### CTP Synthase 1 (CTPS1) deficiency

One study reported two cases with EBV- associated unspecified NHL in patients with CTPS1 deficiency (93).

### IUIS: Defects in Intrinsic and Innate Immunity

#### WHIM (Warts, Hypogammaglobulinemia, Immunodeficiency, and Myelokathexis) Syndrome

WHIM syndrome is caused by gain-of-function defects in the *CXCR4* gene frequently associated with severe neutropenia and variable degree of immunodeficiency. There are two reports (Table 1) of B cell lymphoma (type not specified) in two adult patients with WHIM syndrome (95, 96). Both were associated with EBV infection leading to lymphoproliferation and ultimately lymphoma. Treatment in both cases was with CHOP chemotherapy, and one patient had a CR, while the other patient who presented with intestinal lymphoma had no response and died of intestinal perforation.

## T Cell Lymphomas in PIDs

The incidence of T cell lymphomas is infrequent in PIDDs (Table 2). In this particular literature review, 74 patients were identified through 20 different reports. The types of T cell lymphoma identified included T cell non-Hodgkin lymphoma (TNHL) 36%, T cell lymphoblastic lymphoma (T-LL) 32%, peripheral T cell lymphomas (PTCL) 9%, granulomatous cutaneous T-cell lymphoma (G-CTCL) 4%, anaplastic large cell lymphoma (ALCL) 4%, and T cell-diffuse lymphocytic lymphoma (T-DLL) 1%. Of these cases, 41% (*n* = 30) were male, 10% (*n* = 10) were females and 46% (*n* = 34) did not report gender. The median age at the diagnosis of lymphoma was 13 years (range 0.5–68 years). It is interesting and relevant to note that the cases of T cell lymphomas were mostly observed in patients with predominantly antibody deficiencies or combined immunodeficiencies with associated or syndromic features. Only a single report is available of a patient with a combined immunodeficiency (CID) (Table 2), who developed T-LL and was treated with full dose NHL-BFM95 (Non-Hodgkin Lymphoma-Berlin-Frankfurt-Münster 95) on a phase1 clinical trial (4), and was in CR but subsequently died of sepsis.

**Table 2:** Summary of T cell lymphomas in PIDDs.

**PID**	**References**	**Proposed mechanism**	***N***	**Cancer**	**Age/sex**	**Manifestation/course**	**Treatment**	**Outcome**
**PREDOMINANTLY ANTIBODY DEFICIENCIES**								
CVID	(97)	Immune dysregulation	1	DLL	52/M	RUQ tenderness, early satiety. HS. erythematous skin papules.	Chlorambucil and Prednisone	Poor response, died in 2 years.
CVID	(98)	Immune dysregulation	1	PTCL	57/M	Fever, night sweats, progressive refractory anemia, tender inguinal lymphadenopathy	ProMACE CytaBOM	CR after 6 cycles
CVID	(99)	NS	1	PTCL	32/F	Persistent cytopenia, progressive neurologic disease presenting as a polyradiculopathy with aseptic lymphocytic meningitis	cranial radiation, systemic and intra-thecal chemotherapy	Symptoms progressed. Died in few months.
IgA deficiency	(100)	Immune dysregulation	1	PTCL	24/M	4 years after PID diagnosis: liver failure, abdominal lymphadenopathy, pancytopenia, and recurrent bacterial infections and increasing pulmonary infiltrates	None. Diagnosis on autopsy.	Died of pulmonary failure before diagnosis
XLA	(101)	Chronic antigenic stimulation Immune dysregulation	1	PTCL	21/M	Sustained fever, unresponsive to antibiotics	NR	NR
XLA	(102)	Immune dysregulation	1	GCTCL	33/M	Generalized asymptomatic papulonodular eruption No lymphadenopathy/organomegaly	NR	NR
IgA deficiency	(102)	Immune dysregulation	1	GCTCL	68/M	Progressive generalized papules, plaques, and tumors. Occult IgA def diagnosed after presentation.	cyclophosphamide, methotrexate, etoposide, and dexamethasone	CR followed by recurrence and eventual death in 5 years
CVID	(102)	Immune dysregulation	1	GCTCL	44/M	progressive, asymptomatic red papules and nodules on his trunk and extremities.	Bexarotene Gemcitabine	Relapse and disease progression
IgA deficiency	(41)	NS	2	Pt1: ALCL	11/M		NHL-BFM	CR, LFU +3.5 years
				Pt2: TNHL	2/M		Non-B therapy, induction only	Sepsis during chemotherapy, death after 3 weeks of therapy
CVID	(71)	NS	4/247	4 T-cell	NR	NR	NR	2 died, 2 responded to RT
CVID	(3)		1/416	1 PTCL	NR	NR	NR	NR
APDS	(52)	NS	1/53	ALCL	NR	NR	NR	NR
Hypogammaglobinemia	(41)	NS	1	ALCL	15/M		NHL-BFM	CR, LFU +2.75 years
**COMBINED IMMUNODEFICIENCIES WITH ASSOCIATED OR SYNDROMIC FEATURES**								
NBS	(44)	Chronic antigenic stimulation	5/57	TNHL	NR	NR	NR	15 out of 22 patients (B and T lymphomas) died, most at the beginning of the study, but 7 were clinically stable after early diagnosis and successful treatment
		Chronic antigenic stimulation	2/57	TLBL	NR	NR	NR	
NBS	(45)	NS	21/149	TNHL	NR	NR	NR	44% of all patients were in remission, 54% died due to disease progression
NBS	(103)	EBV associated	1	1 TLBL	10/F	Fever, generalized lymph node enlargement, hepatosplenomegaly and mediastinal mass	BFM90 protocol	Alive 7 years later
NBS	(37)	NS	9	Pt1: TLBL	10/F	NR	BFM90	Died of disease after protocol
				Pt2: TLBL	8/F	NR	BFM90	Died of relapse during maintenance
				Pt3: TLBL	16/M	NR	BFM90	Died of relapse during maintenance
				Pt4: TLBL	12/M	NR	BMF-90 followed by chemotherapy (ICE)	Died of organ failure caused by sepsis
				Pt5: AILT	8/F	NR	BMF-90 followed by individualized chemotherapy	Died of disease
				Pt6: TLBL	7/F	NR	EURO-LB 02 protocol	Alive disease-free
				Pt7: TLBL	3/M	NR	EURO-LB 02 protocol	Alive disease-free
				Pt8: TLBL	9/M	NR	EURO-LB 02 protocol	Alive with disease
				Pt9: TLBL	19/F	NR	EURO-LB 02 protocol	Died of relapse during maintenance
NBS	(38)	NS	6	Pt1: TLBL	18/M	2nd malignancy: AML	EURO-LB 02 protocol	Death because of 2nd malignancy (AML), 3.5 years after diagnosis
				Pt2: TLBL	16/M	NR	EURO-LB 02 protocol	CR 1 year after diagnosis
				Pt3: TLBL	4/M	2nd malignancy: TLBL 14 years after diagnosis	NHL-BFM90	CR after treatment in accordance to GMALL protocol for 2nd malignancy
				Pt4: TLBL	6/F	NR	EURO-LB 02 protocol	CR 2.4 years after diagnosis
				Pt5: TLBL	8/M	NR	ALL-BFM-MR protocol	CR 1.7 years after diagnosis and 1 year after SCT
				Pt6: TLBL	16/M	NR	NHL-BFM95	Death 1 year after diagnosis
NBS	(39)	Defective antitumor immunosurveillance	2/26	Pt1: TLBL	16/M	NR	NR	Death at 18 years
				Pt2: TNHL	24/M	NR	NR	Death at 27 years from sepsis
NBS	(40)	NS	3	Pt1: TLBL	8/F	Cervical LN	NR	Died at 11 years
				Pt2: TLBL	16/M	Cervical LN	NR	Died at 18 years
				Pt3: TLBL	12/M	Cervical LN	NR	Died at 13 years
Ataxia Telangiectasia	(38)	NS	1	Pt1: TLBL	0.5/M	2nd malignancy: B-NHL after 3.5y	NHL-BFM86 modified	Death of second malignancy
Ataxia Telangiectasia	(41)	NS	1	TNHL	0.5/M	2nd malignancy: low-grade B-NHL	Non-B therapy, induction with 50% dosage, then reduced maintenance; omission of therapy after 7 months from severe toxicity	Reached CR initially; died of 2nd malignancy after 3.75 years
NBS	(4)	Defective DNA repair	3	Pt 1: TLBL	5/M	NR	NHL-BFM95, phase I protocol, nonmyeloablative conditioning, HSCT	CR 1, alive disease-free for 1.4 years
				Pt 2: PTCL	16/M	NR	CHOP–CHOP–ICE– DHAP–DHAP– DexaBEAM	Died of disease at 5 months after the diagnosis
				Pt 3: TLBL	14/M	NR	NHL-BFM95, protocol I, full dosage	Death of sepsis in PR after 7 weeks of therapy
**IMMUNODEFICIENCIES AFFECTING CELLULAR AND HUMORAL IMMUNITY**								
CID	(4)	Impaired Immune function	1	TLBL	8/M	9	NHL-BFM95, full dosage	Death of sepsis in CR 2 at 2 years after the diagnosis
**DISEASES OF IMMUNE DYSREGULATION**								
CD27 deficiency	(88, 104)	EBV associated	1	Pt1: TCL	16/M	Oral ulcers, uveitis, chronic EBV viremia, and EBV-related LPD progressing to T-cell lymphoma	Rituximab, R-CHOP, cord HSCT	Alive
Hypergammaglobinemia	(41)	NS	1	PTCL	2/F		Non-B therapy, induction, followed by maintenance	Still on maintenance therapy in PR for 2 years

*AILT, (angioimmunoblastic T cell lymphoma); AML, (acute myloid leukemia); CID, (combined immunodeficiency); CVID, (Common variable immune deficiency); DLL, (diffuse lymphocytic lymphoma); CR, (complete remission); F, (female); GCTCL, (granulomatous cutaneous T-cell lymphoma); HS, (hepatosplenomegaly); LN, (lymph nodes); M, (male); NR, not reported; NBS, Nijmegan breakage syndrome; NHL-BFM, Non-Hodgkin Lymphoma-Berlin-Frankfurt-Münster; NS, not specified, PR, partial remission; ProMACE, prednisone, doxorubicin, cyclophosphamide, etoposide, methotrexate, leucovorin; Pt, patient; PTCL, peripheral T cell lymphoma; R-CHOP, Rituximab + cyclophosphamide, doxorubicin, vincristine, and prednisone; RUQ, right upper quadrant; TLBL, T lymphoblastic lymphoma; XLA, X-linked agammaglobulinemia; TCL, T cell lymphoma*.

**Table 3:** Summary of unspecified lymphoma in PIDDs.

**PID**	**Reference**	***N***	**Proposed mechanism**	**Cancer**	**Age/sex**	**Manifestation/course**	**Treatment**	**Outcome**
XMEN^*^	(89)	1/3	EBV associated	Lymphoma	45/M	NR	NR	Died at the age of 45
ADA deficiency	(105)	1	Not specified	Lymphoma	NR/M	NR	HSCT	Died
CVID	(54)	1	Not specified	Malignant lymphoma	30/M	NR	NR	NR
CVID	(65, 66)	1/117	Not specified	Undifferentiated lymphoma	63/M	Bone marrow	Plasmapheresis, CHOP, M-BACOD	Died 2 years later
CVID	(106)	71/2212	Not specified	Lymphoma	NR	NR	NR	NR

CHOP, (cyclophosphamide, doxorubicin, vincristine, and prednisone); CVID, common variable immune deficiency; EBV, Epstein-Barr virus; F, female; HCT, hematopoietic cell transplantation; M, male; M-BACOD, (methotrexate, doxorubicin, cyclophosphamide, vincristine, dexamethasone); NR, not reported. *EBV positive.

### IUIS: Diseases of Immune Dysregulation

#### CD27 Deficiency

A 16 year old male presented with oral ulcers, uveitis, chronic EBV viremia, and EBV-related lymphoproliferative disorder (LPD) progressing to T-cell lymphoma was treated with rituximab, R-CHOP, and subsequently a cord blood HCT, and the patient was alive at last follow-up (104).

### IUIS: Immunodeficiencies Affecting Cellular and Humoral Immunity

#### CID

Only a single report of a patient with a combined immunodeficiency (CID) is reported (Table 2) who developed T-LL and was treated with full dose NHL-BFM95 on a phase1 clinical trial, and was in CR but subsequently died of sepsis (4).

### IUIS: Predominantly Antibody Deficiencies

In patients with humoral immune defects, there were 10 studies with 17 patients reported (Table 2). The types of T cell lymphomas observed were PTCL (29% *n* = 5), G-CTCL (18%, *n* = 3), ALCL (18%, *n* = 3), T-DLL (5% *n* = 1), and TNHL (5%, *n* = 1). The median age at diagnosis was 32 years (range: 2–68 years) with a distribution of males (59%, *n* = 10), females (6%, *n* = 1) and NS (35%, *n* = 6).

#### Common Variable Immunodeficiency (CVID)

Of the patients with humoral defects, 53% (*n* = 9) had CVID across six studies, and the main types of T cell lymphomas included were PTCL, T-DLL, G-CTCL, and 4 unspecified T-cell lymphomas (3, 71, 97–99, 102). The treatment modalities used include Prom ACE (prednisone, doxorubicin, cyclophosphamide, etoposide, methotrexate, leucovorin), CytaBOM (cytarabine bleomycin, vincristine, methotrexate), radiation, systemic and intra-thecal chemotherapy. A single CVID patient presented with G-CTCL, which relapsed and progressed after treatment with bexarotene and gemcitabine. Another CVID patient developed T-DLL and had a poor response to chlorambucil and prednisone and succumbed to disease within 2 years.

#### Selective IgA Deficiency (sIgAD)

As with B cell lymphoma, there were three reports of four patients with sIgAD who developed T cell lymphoma (G-CTCL, PTCL, ALCL, TNHL) (41, 100, 102). All were males, and the diagnosis was made by autopsy in one patient, while the diagnosis of occult IgA deficiency was made in the second patient after the development of malignant disease. Though this patient was treated with chemotherapy with apparent CR, there was subsequent relapse of disease and mortality within 5 years. In patients classified as having sIgAD and malignant disease, it remains an open question as to whether there was a more severe underlying immunological defect that was not identified at the time.

#### Hypogammaglobinemia

Seidemann et al. reported a case of 15-year-old male with hypogammaglobulinemia who developed ALCL (41). Complete remission was achieved on treatment with chemotherapy. Last follow up was almost three years after diagnosis. A molecular etiology for the hypogammaglobulinemia was not available in this patient.

#### XLA

In the 2 patients reported with XLA, the types of T cell lymphoma observed were G-CTCL and PTCL (101, 102).

### IUIS: Combined Immunodeficiencies With Associated or Syndromic Features

In this category, we identified nine studies with 54 patients. Median age was 10 years (0.5–24). Similar to other categories with T cell lymphomas, males had a higher percentage (33%, *n* = 18) compared to females (15%, *n* = 8) and 52% (*n* = 28) were NS. TNHL (52%, *n* = 28) was the most common type of TCL followed by TLBL (44%, *n* = 24).

#### Nijmegen Breakage Syndrome (NBS)

Similar to B cell lymphomas (BCL) in NBS, there are reports of T cell lymphoma (TCL) in this group of patients. In Table 2, there are eight studies with 52 patients, all under the age of 20 (4, 37–40, 44, 45, 103). The underlying mechanism for development of malignant disease is the same as for BCL and related to defects in DNA repair. The commonly used chemotherapy protocols in these patients included NHL-BFM-90, NHL-BFM-95, and EURO-LB 02.

#### AT

We identified two studies reporting two cases of TCL in AT patients (38, 41). A 6-month-old boy was diagnosed with TLBL and treated with a modified NHL-BFM 86 regimen. He developed B-NHL at the age of four leading to death. The second patient was also a 6-month-old boy who developed T-NHL and achieved complete remission initially, but like the first patient died at the age of four due to a second malignancy (B-NHL).

## Discussion

In this section, we discuss the mechanisms responsible for lymphomagenesis in the various inborn errors of immunity and provide an overview of the treatment.

## Defects in Immune Responses That Predispose to Lymphomagenesis in PIDDs

**Figure 3:**
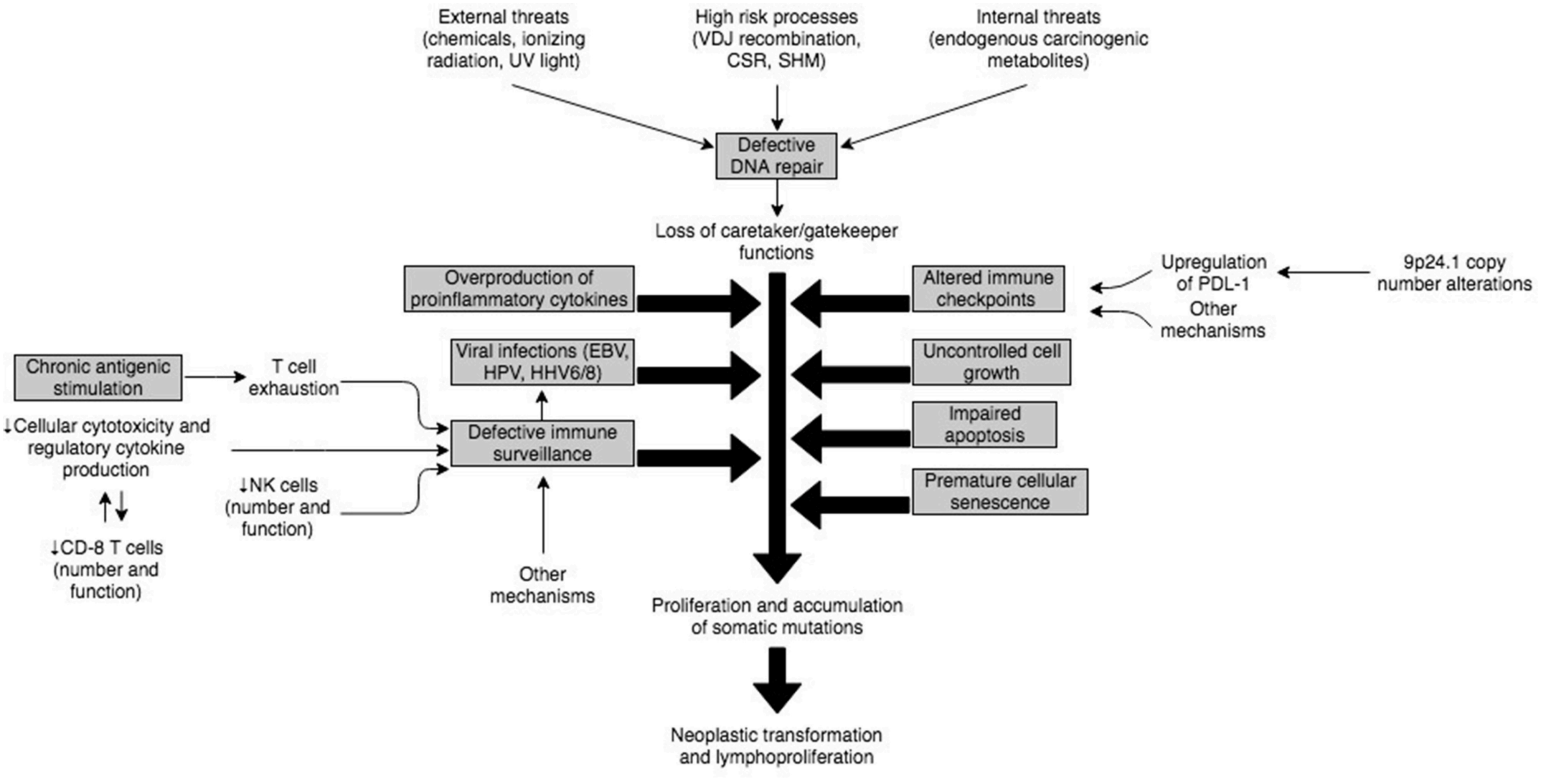
Illustration of potential interplay of mechanisms implicated in pathogenesis of malignancies in PIDs.

The complex immune mechanisms and their interplay that predisposes to neoplastic transformation of B or T cells and development of lymphomas in PIDD patients has not been fully elucidated. However, it is expected that the etiology in most cases is multifactorial and related to a dynamic regulation of immune response and environmental triggers (Figure 3). An underlying intrinsic susceptibility to DNA damage in some of these PIDDs, may provide a substrate for uncontrolled cell growth, impaired apoptosis of damaged cells, premature cellular senescence, all of which may be compounded by increased antigenic stimulation of adaptive lymphocytes by viruses, such as EBV (112). This uncontrolled stimulation in the setting of altered immune checkpoints and immune dysregulation characterized by T cell exhaustion, defective anti-tumor immune surveillance and overproduction of inflammatory cytokines provides a fertile setting for neoplastic transformation and lymphoproliferation (Figure 3).

### Defective DNA Repair

DNA integrity is constantly challenged at various levels as part of physiological processes. In lymphocytes, there is particular susceptibility specifically related to intrinsic mechanisms that are part of the immune diversity generation apparatus, such as V(D)J recombination, class switch recombination (CSR), and somatic hypermutation (SMH) (113). These intrinsic “stress points” coupled with other factors, including exposure to internal mutagens (e.g., endogenous metabolites) and external factors such as ultraviolet rays, ionizing radiation and chemicals (107) sets the stage for development of dysregulated cellular proliferation in B or T cells. Therefore, it is not surprising that normally there is a highly conserved DNA repair system orchestrated by a network of enzymes, which constantly assesses and detects DNA damaging lesions, modifies or removes damaged DNA, and reconstitutes the integrity of DNA through nucleotide resynthesis and ligation (114). When these checks and balances fail in the context of monogenic defects, it promotes the development of lymphoid tumors (113). Patients with premature cellular senescence related to shortened telomeres not only have an intrinsic susceptibility to DNA damage-inducing stimuli but also may have impaired apoptosis of damaged cells, which in turn may promote lymphoproliferation.

### Role of Viral Infections and Defective Immune Surveillance, and Immune Dysregulation

Several of the documented PIDDs increase susceptibility to viral infections such as EBV, human papilloma virus (HPV), human herpes virus-6 (HHV-6), HHV-8, human T-cell lymphotropic virus (HTLV), Kaposi sarcoma–associated virus (KSV), and other viruses due to defective immune surveillance and immune dysregulation (115). Among these viruses, EBV represents the biggest threat because of its high prevalence (95%) and ability to transform epithelial cells, B cells, T cells and NK cells (116).

In the majority of cases, EBV virions entering through the oro-pharynx infect epithelial cells and B cells via the CD21 receptor. From a host immune system standpoint, NK cells and antigen-specific CD8+ T-cells are the main defense against primary EBV infection. Therefore, if EBV-infected B cells escape the cellular immune response, it can provoke an inflammatory outburst, resulting in cellular hyperproliferation and abnormal cell survival eventually leading to EBV-associated lymphoproliferative disorders (EBV-LPDs). EBV-LPDs consist of virus-associated hemophagocytic syndrome, non-malignant and malignant B-cell LPDs including non-Hodgkin and Hodgkin's types of B lymphomas and rarely EBV-positive T/NK cell lymphoma (117). The critical role of NK and T cells is demonstrated by the fact that combined B and T cell deficiency PIDDs account for approximately 2/3rd of EBV-associated LPDs in PIDDs, whereas defects in innate immunity do not significantly increase the risk of EBV-associated LPDs (116). Thus, underlying genetic defects in *SH2D1A* (SAP–XLP-1), *ITK, MAGT1, CD27, CD70, CTPS1, RASGRP1, CORO1A* and others account for the majority of EBV-LPDs.

The lack of adequate immune surveillance in the context of immune dysregulation is not uncommon in PIDDs, and though these cannot be exhaustively discussed here, failure of both innate (NK cells) and adaptive (T cells) immune cellular mechanisms facilitate viral escape and subsequent viral transformation of lymphocytes with poor cytotoxic clearance of virally-infected cells (89, 117). On the other hand, in certain other PIDDs, for example, the IL-10 receptor defects, there is production of pro-inflammatory cytokines due to the lack of IL-10-based regulation, and NFκB activation, with defective intra-tumoral CD8+ T cell immune surveillance and impaired cytotoxicity. This results in B cell proliferation and accumulation of somatic mutations (94, 111). Therefore, this is associated with a non-EBV-associated lymphoproliferation.

### Chronic Antigenic Stimulation

Patients with PIDDs are more likely to develop persistence of antigens, and therefore ongoing stimulation of effector immune cells. T and B cells respond to external stimuli (antigens) and subsequently eliminate non-self-antigens (pathogen-infected and/or transformed cells). Sustained antigenic stimulation, in the context of cellular and systemic immune compromise, is likely to increase T cell exhaustion with progressive loss of cytokine secretion (IL-2, TNF-<), impairment of IFN-γ production, and in extreme cases premature apoptosis of CD8+ T cells (118). This quantitative and qualitative loss of the CD8+ T cell immune response against tumor cells eventually results in the development of lymphomas, and another plausible mechanism for tumor development in PIDDs.

### Altered Immune Checkpoints

One of the primary mechanisms by which tumors escape the immune system is by engaging immune checkpoints (119), which is why immune checkpoint inhibitors (ICIs) were approved for the treatment of cancers and are now used in 14 different types of cancer (120). This issue is relevant to the lymphomas, which develop in PIDDs as it has been shown that PDL-1 protein is overexpressed in most EBV-associated tumors (121). Further, copy number variations (CNVs) at 9p24.1 have also been reported, and this is a locus, which harbors PD-1 (122). However, only one of two studies found amplifications of chromosome 9p via array CGH (comparative genomic hybridization) in EBV+ DLBCL (123). These findings raise the possibility that EBV+ DLBCL evades immune surveillance by selectively targeting the PD1/PDL pathway. Therefore, there may be some value to using immune checkpoint inhibitors in PIDDs for the treatment of lymphoma, and this raises the need for clinical trials in this area.

## Treatment of B and T Cell Lymphomas in PIDDs

### Clinical Presentation, Diagnosis and Staging

PIDD patients often present with advanced disease, and extranodal sites of disease, including bone marrow, liver, and spleen, and the presence of B-symptoms is quite common. There is no difference in how the diagnosis of lymphoma is established, whether in in PIDDs or non-intrinsic lymphoid malignancies, and it usually involves tissue biopsy, most frequently of lymph nodes.

Clinical evaluation should include standard workflow of history and physical, complete blood count with differential, liver and kidney function and baseline pulmonary function test for adult patients, if they will undergo bleomycin-containing regimens in the context of HL. Additional work-up to assess the degree of immune impairment, based on underlying genetic defect is essential for this patient population, and should include evaluation of radiosensitivity (especially if the genetic defect is known to predispose to this) so that appropriate treatment regimens can be formulated. Principles of diagnostic imaging may be extrapolated from imaging recommendations for Hodgkin's lymphoma in HIV patients, which suggests 18FDG-PET with integrated or concurrent CT (computerized tomography) of the neck, chest, abdomen, and pelvis. Since 18FDG-PET imaging has not been validated in this setting, it is unclear whether a bone marrow biopsy can be omitted. The interpretation of 18FDG-PET scan be challenging in presence of concurrent infections, which is not uncommon in these patients (124).

### Treatment Options

While there are no specific treatment options for lymphoma in PIDD patients, radiation-based therapies should be avoided in patients with known genetic defects that predispose to radiosensitivity. Most frequently standard treatment options are based on type of lymphoma, such as B or T cell, indolent, aggressive or very aggressive and patient-related characteristics such as age, comorbidities, immune status, and degree of immune compromise. While there are no large or randomized studies to confirm this, small series and case reports show that response to the treatment and prognosis is inferior in PIDD patients when compared to non-PIDD patients, largely related to their inability to tolerate standard chemotherapy, and susceptibility to life-threatening infections (25). HCT remains the only viable curative option for many of these diseases, though its efficacy of HCT is variable across different PIDDs. For example, the role of HCT in severe combined immune deficiencies (SCID) is well established and it has been consistently replicated (125). HCT is not a standard treatment of choice in CVID, especially those without a molecular defect, though it may be warranted in certain cases depending on the presentation and associated co-morbidities. In circumstances, of a specific molecular diagnosis replacing or clarifying the underling CVID, HCT may be the most optimal long-term treatment of choice, even though more targeted therapies may be available. HCT for CVID was reported in a multicenter experience for 25 patients transplanted at 14 centers in Europe, the US, and Japan, and though it may be considered for lymphoma in this context, the risk of transplant-related mortality (TRM) should be clearly weighed against the potential benefits (126). Several retrospective studies have reported outcomes of HCT as across a range of PIDDs, including SCID (127), and Wiskott Aldrich Syndrome (128). Only case reports or small case series are available for successful treatment in patients with other PIDDs such as GATA2 haploinsufficiency, IL-10 receptor or XIAP deficiencies. Despite the limited experience, data strongly favors the early use of HCT in the setting of IL-10 receptor deficiency (111). Recently, a retrospective case series of 29 adult patients with a variety of PIDDs (mean age: 24 years, range: 17–50 years) treated with reduced intensity conditioning (RIC)-HCT was published. This study reported an overall survival of 85.2% at 3 years (129). These data provide support for moving forward with HCT in adult PIDD patients. As such, in addition to the treatment of lymphomas, HCT can be lifesaving in patients with PIDDs who develop very severe complications, including susceptibility to life-threatening infections, bone marrow failure, autoimmune and autoinflammatory diseases, other malignancies, and hemophagocytic syndrome. Despite all these potential benefits, it remains to be ascertained as to whether early HCT can reduce the risk of developing lymphoma in these PIDD patients in the long term.

There is no consistent evidence to support the use of newer immunotherapies for treatment of lymphoma in PIDDs but these therapies hold significant mechanistic promise for personalized, chemotherapy-free treatments. Potential immunotherapy options include monoclonal antibody-based immunotherapy (e.g., Rituximab, Obinutuzumab, Epratuzumab), conjugated antibodies (Brentuximab Vedotin), Bi-Specific T cell engaging (BiTE) antibodies (Blinatumumab), Anti-PD1(Pembrolizumab, Nivolumab), Anti PDL-1 (Atezolizumab and Darvalumab), and anti-CTLA4 checkpoint inhibitors (e.g., Ipilimumab). The role of adoptive T cell therapies such as chimeric antigen receptor (CAR) T-cells remains uncertain at this point (130). While these targeted therapies may have value, they may also pose an increased risk as a result of immune manipulation in the context of an underlying immune deficiency or immune dysregulation.

While there are no reports for successful use of immune checkpoint inhibitors or BiTE antibodies, complete remission with Rituximab and Brentuximab Vedotin was reported in an adult female patient with CVID -associated classic HL, while two other cases of pediatric CVID-associated HL succumbed to severe infection related to chemotherapy (61).

## Conclusions

Though this is not a comprehensive summary of malignancies in PIDDs, or even lymphoproliferative disease in this area, this review summarizes the Medline-indexed published reports of B and T lymphomas in patients with PIDDs. This report highlights the diversity of malignant lymphoproliferative disorders in setting of PIDDs, and its associated challenges of diagnosis and treatment. The pathological classification and nomenclature for the lymphoid malignancies with variably reported and postulated underlying mechanisms were inconsistent and inadequate for many of these published reports. A wide range of treatment options were utilized, and response rate was highly variable suggesting an empirical approach rather than a systematic and tailored treatment regimen, based on underlying genetic defect, and degree of immunological impairment. HCT and gene therapy (where available) remains the best treatment option for many, but not all of these patients, and should be promptly initiated after diagnosis, particularly in some conditions, such as SCID, WAS, IL10 receptor deficiency among others. HCT, as a therapeutic option, remains significantly under-utilized in adult patients, likely related to inadequate awareness among adult hematologists, and these patients may benefit from increased utilization of HCT in appropriate settings. We highlight the significant need of unifying nomenclature, pathological analysis, and assessment of mechanisms of lymphomagenesis in these patients to develop better and more personalized treatment regimens. Also, there is a considerable urgency to conduct clinical trials to develop evidence-based treatment plans that considers the underlying immunodeficiency rather than using approaches extrapolated from non-PIDD settings (131). It is recognized that there has been a recent effort to standardize classification and nomenclature in immunodeficiency-associated malignant LPDs, but this is largely focused on secondary immunodeficiency disorders, in contrast to primary, though presumably some of the same standards could be applied (132), but this should be undertaken specifically for PIDDs, to fully understand the similarities and differences in pathology.

## Author Contributions

All authors designed the study. IR and WF performed the literature search, conducted data extraction of relevant studies, and wrote the first draft of the manuscript. MP and RA critically reviewed the literature search and made revisions in the manuscript. All authors read and approved the final version of the manuscript.

### Conflict of Interest Statement

The authors declare that the research was conducted in the absence of any commercial or financial relationships that could be construed as a potential conflict of interest.
